# Secretory Acid Sphingomyelinase in the Serum of Medicated Patients Predicts the Prospective Course of Depression

**DOI:** 10.3390/jcm8060846

**Published:** 2019-06-13

**Authors:** Christiane Mühle, Claudia Johanna Wagner, Katharina Färber, Tanja Richter-Schmidinger, Erich Gulbins, Bernd Lenz, Johannes Kornhuber

**Affiliations:** 1 Department of Psychiatry and Psychotherapy, Friedrich-Alexander University Erlangen-Nürnberg (FAU), Schwabachanlage 6, D-91054 Erlangen, Germany; claudia.wagner@uk-erlangen.de (C.J.W.); katharina.faerber@gmx.de (K.F.); tanja.richter-schmidinger@uk-erlangen.de (T.R.-S.); bernd.lenz@uk-erlangen.de (B.L.); johannes.kornhuber@uk-erlangen.de (J.K.); 2 Department of Molecular Biology, University of Duisburg-Essen, D-45259 Essen, Germany; erich.gulbins@uni-due.de; 3 Department of Surgery, University of Cincinnati, Cincinnati, OH 45267-0558, USA

**Keywords:** acid sphingomyelinase, anxiety, ceramide, course of depression, lipids, major depression, predictive biomarker, quality of life, sphingolipid metabolism

## Abstract

Major depressive disorder (MDD) is a highly prevalent and devastating psychiatric illness with strong individual and societal burdens. However, biomarkers to improve the limited preventive and therapeutic approaches are scarce. Multilevel evidence suggests that the pathophysiological involvement of sphingolipids particularly increases the levels of ceramides and the ceramide hydrolyzing enzyme, acid sphingomyelinase. The activity of secretory acid sphingomyelinase (S-ASM) and routine blood parameters were determined in the serum of patients with current (unmedicated *n* = 63, medicated *n* = 66) and remitted (*n* = 39) MDD and healthy subjects (*n* = 61). Depression severity and anxiety and their 3-weeks prospective course of treatment were assessed by psychometric inventories. S-ASM activity was not different between the four groups, did not decrease during treatment, and was not lower in individuals taking medication that functionally inhibited ASM. However, S-ASM correlated positively with depression severity only in remitted patients. High enzyme activity at inclusion predicted milder clinician-evaluated and self-rated depression severity (HAM-D, MADRS, BDI-II) and state anxiety at follow-up, and was related to stronger improvement in these scores in medicated patients. S-ASM was strongly and contrariwise associated with serum lipids in unmedicated and medicated females. These findings contribute to a better understanding of the pathomechanisms underlying depression and the development of clinical strategies and biomarkers.

## 1. Introduction

Major depressive disorder (MDD), with a lifetime prevalence of more than 10%, is a severe mood disorder and a leading cause of disability worldwide, with substantial economic consequences for the society. A major depressive episode (MDE) is characterized by a combination of affective, social, and somatic symptoms, such as anhedonia, anxiousness, low self-esteem, feeling of worthlessness, lack of concentration and motivation, neglect of hobbies and interests, social isolation, fatigue, sleep disturbances, and changes in appetite or weight. MDD strongly impairs psychosocial functioning and quality of life.

Depression is associated with an increased mortality rate that exceeds 1.65 times that of the general population [1]. Most suicides are committed by depressed individuals [2], but clinically relevant predictors are still lacking [3,4]. Despite effective treatments for depression, the disease burden of depression is only reduced by 10–30% by curative interventions, which is largely due to limitations in diagnosis, and availability of and adherence and response to appropriate treatment [5].

Despite tremendous efforts, the lack of reliable markers of depression is still a major obstacle to the diagnosis of endophenotypes and individualized treatment of MDD. Clinician-administered scales for depression severity, as well as self-report inventories, are mainly used to diagnose and classify the severity of depression and to monitor its course during therapy. Both approaches are limited by rater and/or patient biases in addition to the heterogeneous presentation of the disease. Therefore, objective biomarkers for depression per se, its severity, and its course are needed.

Although the pathophysiological mechanisms of depression are not fully understood, there are various and partially overlapping theories, including the monoamine, stress, immune-inflammation, and neuroplasticity hypotheses [6], which still cannot fully explain the observed effects [7]. Multiple evidence exists for the dysregulation of the sphingolipid metabolism in depression, which has been similarly found to be responsible for other psychiatric disorders in human studies and animal models, such as alcohol dependence, anxiety, and schizophrenia [8].

Sphingolipids not only serve as a membrane component to form a physical barrier and as ligands on their own, but also influence the local composition and fluidity of the plasma membrane and thereby the localization and activity of proteins involved in receptor-mediated signaling [9]. The balance between ceramide with its pro-apoptotic function and its anti-apoptotic metabolite sphingosine-1-phosphate is of particular relevance for various physiological and pathophysiological conditions [10]. Ceramide levels are regulated by sphingomyelinases and ceramidases, de novo biosynthesis, and the salvage pathway [11].

Acid sphingomyelinase (ASM, EC 3.1.4.12, [12]) catalyzes the hydrolysis of the abundant membrane lipid sphingomyelin into ceramide and phosphorylcholine in the lysosome at an acidic pH (L-ASM). A second secretory form (S-ASM) encoded by the same gene (*SMPD1*) is located extracellularly and differs in several characteristics, including a longer in vivo half-life [13]. Considerably decreased serum or plasma S-ASM activity has been observed only for gene variations or mutations of *SMPD1*, such as in patients with the lysosomal storage disorder Niemann-Pick disease [14], and is thus considered as a trait marker. In contrast, elevated enzyme activities as a state marker have been noted in a variety of common human diseases and are thought to result from an increased enzyme activation (e.g., induced by oxidative stress) or its enhanced release (e.g., caused by lipopolysaccharide and cytokine stimulation). Due to its stability, this enzyme might also supply information on past inflammatory processes and thus serve as a potential clinical biomarker [15].

The first evidence for the involvement of ASM in depression was identified by the observation of elevated enzyme activities in peripheral blood mononuclear cells of patients with MDE and a correlation of this increase with depression severity [16]. In cultured patients’ cells, the ASM activity decreased by treatment with classical antidepressants imipramine and amitriptyline [16]. Several further small drug-like molecules, many of them licensed for medical use in humans, including other antidepressant drugs, were found to functionally inhibit ASM and thus termed FIASMAs (Functional Inhibitor of Acid SphingoMyelinAse) [17,18]. Among these, amitriptyline and fluoxetine also reduced ASM activity and ceramide levels in mice coincident with increased neuronal proliferation, maturation and survival, and reduction of stress-induced depressive behavior [19]. The slow accumulation of these lipophilic, weakly basic drugs by acid trapping in the lysosome is thought to be responsible for the therapeutic latency of antidepressants [20].

Further human studies have identified elevated plasma ceramides in patients with major depression, regardless of their Alzheimer’s dementia status [21], and an association of plasma sphingomyelin levels with depressive symptoms in a large family-based study [22]. Combat veterans with post-traumatic stress disorder presented with a 1.6-fold increase in plasma S-ASM activity and a 2-fold higher level of sphingosine-1-phosphate [23].

In animal models, lipidomic profiling of the effect of chronic unpredictable stress in rats revealed an increase in ceramide and a decrease in sphingomyelin in specific brain regions [24]. Chronic psychosocial stress was also associated with an increase in serum S-ASM activity in male mice housed with a larger dominant male versus single housed controls [25]. The sphingolipid mechanism has further been proposed for mild depression induced by the extinction of learned behavior, which is accompanied by emotional behaviors indicative of frustration, anxiety, and despair. Efficient re-learning of conditioned behavior in rats correlated with a decrease in ASM activity in the dorsal hippocampus [26].

MDD and alcohol dependence share a high comorbidity, which can partially be explained by the use of alcohol as a way of self-medication for psychiatric problems, including depression. In alcohol dependence, the activity of L-ASM in peripheral mononuclear blood cells is significantly increased and decreases during withdrawal treatment [27]. The same effect is considerably larger for the secretory form of the enzyme [28,29,30]. Further evidence for a link between ASM and depression has been found in a mouse model. Genetically induced overexpression of ASM resulted in depression-like behavior, enhanced the consumption of alcohol, and facilitated the establishment of drug memory. Free-choice alcohol consumption, but not sole injection of alcohol, reversed depression-like behavior in transgenic ASM mice and restored ASM homeostasis [31].

In our large and sex-balanced cohort of patients with current MDE, matched healthy controls, and patients with remitted MDD, we aimed at characterizing the potential of the readily quantifiable activity of peripheral S-ASM as a biomarker for depression, its severity, and prospective course. Based on the literature, we tested the following hypotheses: (1) There are group differences with S-ASM activity in unmedicated depressive patients > medicated depressive patients > remitted depressive patients ≥ healthy controls, with the latter comparison depending on whether S-ASM would be a state or trait marker of depression and lower activities in individuals taking FIASMAs. (2) A decrease in S-ASM activity during the treatment of depressed patients is associated with an amelioration of depressive symptoms. (3) There is a positive correlation of S-ASM activity with depression severity. Moreover, we performed further exploratory analyzes to find associations of this enzyme with related traits, such as anxiety and quality of life, as well as laboratory parameters recently found to be altered in depression.

## 2. Materials and Methods

### 2.1. Study Description

We used samples and data from the CeraBiDe (“Ceramide-associated Biomarkers in Depression”) study [32], which was approved by the Ethics Committee of the Medical Faculty of the Friedrich-Alexander University Erlangen-Nürnberg (FAU, ID 148_13 B, 17.07.2013). The recruitment was conducted between 01/2014 and 01/2017 in accordance with the ethical principles of the World Medical Association (sixth revision of the Declaration of Helsinki, Seoul 2008) and the International Conference on Harmonization Guidelines for Good Clinical Practice (1996). All participants provided written informed consent. Depressed patients were recruited from in- and outpatients of the Department of Psychiatry and Psychotherapy at the University Hospital Erlangen and from further interested individuals fulfilling the inclusion criteria. Healthy control subjects were local citizens attracted via e-mails, flyers, letters, local newspapers, and internet advertisement, and underwent a multi-step screening procedure to exclude severe somatic and psychiatric morbidity (with the exclusion of nicotine dependence). We included 129 patients with a current MDE (63 without any antidepressants for at least 2 weeks, 66 subjects taking antidepressants in a stable regime for at least 2 weeks), 61 healthy control subjects, and 39 patients with a remitted MDD (i.e., individuals with a first MDE at the age of less than 60 years and no depressive episode during the preceding 12 months). One further female unmedicated patient, part of the original CeraBiDe study, was excluded from this project due to the unavailability of S-ASM activity. The study sample characteristics are provided in Table 1 with separate data for females and males in Supplementary Tables S1 and S2.

The inclusion criteria were age 18–75 years, and BMI 18.5–35.0 kg/m². The exclusion criteria were severe physical illness (e.g., cancer, diabetes), autoimmune disorders, pregnancy, breastfeeding, and use of anti-inflammatory drugs or corticosteroids within the last 7 days (see [32] for details). All participants were screened using the structured clinical interview for DSM-IV (SKID-I). Of the 129 patients with a current MDE, 59 unmedicated and 60 medicated patients participated in a direct follow-up (21 and 19 days post inclusion (median), with interquartile ranges of 17–28 and 15–24, respectively). All patients received treatment as usual during the observation period, which meant an adjustment of psychotropic drug administration for some individuals (see [32] for details). Whole blood, behavioral scores, and other parameters were collected at the time of recruitment and follow-up.

### 2.2. Psychometric Scales

The depression severity was quantified using the 17-item Hamilton Depression Rating Scale (HAM-D, [33]) and the 10-item Montgomery and Åsberg Depression Rating Scale (MADRS, [34]) assessed by clinicians, as well as the 21-item Beck Depression Inventory II (BDI-II, [35]) for self-evaluation. The State-Trait Anxiety Inventory (STAI) was applied to examine self-reported trait and state levels of anxiety with 20 items each [36].

The SF-12 instrument was used as a shortened version of the SF-36 to quantify the self-reported health-related quality of life [37]. The SF-12 contains twelve questions that measure eight health domains subdivided into physical health (general health, physical functioning, physical role, and body pain for the physical component score) and mental health (vitality, social functioning, emotional role, and mental health for the mental component score). It provides two composite scales normalized to the German population where higher values indicate the better health-related quality of life.

### 2.3. Blood Analysis

The blood samples were collected after overnight fasting in the morning for all individuals to minimize circadian effects. The serum vials were centrifuged (10 min and 2000× *g* at room temperature), and serum was aliquoted and placed into storage at −80 °C for later S-ASM activity assays within 2 h after drawing blood. The routine laboratory parameters were quantified at the Central Laboratory of the Universitätsklinikum Erlangen, Germany (DIN EN ISO 15189 accredited) from separately collected vials.

### 2.4. Determination of S-ASM Activity

The activity of S-ASM was determined using the fluorescent substrate BODIPY-FL-C12-SM (N-(4,4-difluoro-5,7-dimethyl-4-bora-3a,4a-diaza-s-indacene-3-dodecanoyl) sphingosyl phosphocholine, D-7711, Invitrogen, Carlsbad, CA, USA/Life Technologies, Grand 15 Island, NY, USA), as described previously [38]. Briefly, the reaction was performed in 96-well polystyrene plates with 58 pmol sphingomyelin in a reaction buffer totaling 50 µL in volume (200 mM sodium acetate buffer (pH 5), 500 mM NaCl, 0.2% Nonidet P-40 detergent, and 500 µM ZnCl_2_). The reaction was initiated by the addition of 6 µL of a 1:10 dilution of serum in physiological (154 mM) NaCl solution. After incubation at 37 °C for 24 h, the reactions were stopped by freezing at −20 °C and stored until further analysis. For direct chromatography, 1.5 µL of the reaction was spotted directly without further purification on silica gel 60 thin layer chromatography plates (ALUGRAM SIL G, 818232, Macherey-Nagel, Düren, Germany). The product and uncleaved substrate were separated using ethyl acetate with 1% (v/v) acetic acid as a solvent. The spot intensities were detected on a Typhoon Trio scanner and quantified using the ImageQuant software (GE Healthcare Life Sciences, Buckinghamshire, UK). All enzyme activity assays were carried out with four replicate dilutions of each sample, using the same lot of reagents and consumables, and performed by a single operator.

### 2.5. Statistics

The data were analyzed using IBM SPSS Statistics Version 21 for Windows (SPSS Inc., Chicago, IL, USA) and visualized by GraphPad Prism 7.00 (GraphPad Software Inc., San Diego, CA, USA). Continuous data are presented as the median and interquartile ranges in tables as calculated by the custom tables function of SPSS. In case of missing data points (percentage indicated in Table 1), study subjects were excluded from the specific analyses. Spearman’s method was employed to evaluate bivariate correlations. The group differences were tested using the Mann–Whitney U-test for continuous or the χ^2^ test for nominal variables. The differences between inclusion and follow-up were assessed by the Wilcoxon signed-rank for related samples. *P* values less than 0.05 for two-sided tests were considered nominally statistically significant. We have not corrected *p* values for multiple testing and thus report nominal *p* values. For the primary hypotheses, female and male subjects were analyzed together. Subsequently, an explorative sex-specific analysis was performed because of the well-established sex differences in depression [39,40].

## 3. Results

### 3.1. Cohort Characteristics

The groups of unmedicated and medicated patients and healthy controls were sex-balanced. It proved difficult to recruit an equivalent number of males with remitted MDD leading to a sex imbalance in this group (Table 1). Patients were slightly older, but the difference was only nominally significant for remitted patients. There was no significant difference in education level. Patients on medication had a higher body mass index (BMI) compared to healthy controls and compared to unmedicated patients, possibly due to the side effects of antidepressants. High scores on all three depression scales (Hamilton Depression Rating Scale (HAM-D), Montgomery and Åsberg Depression Rating Scale (MADRS), and Beck Depression Inventory II (BDI-II)) demonstrate the medium to high depression severity of included patients with current MDE. These scores decreased from inclusion to follow-up (at a median of 3 weeks later) for both groups and all scales (all *p* < 10^−7^) proving the effectiveness of therapy. Remitted patients showed low depression scores, but these were still higher than for the healthy controls.

### 3.2. Group Differences and Time Course for S-ASM Activity

**Figure 1:**
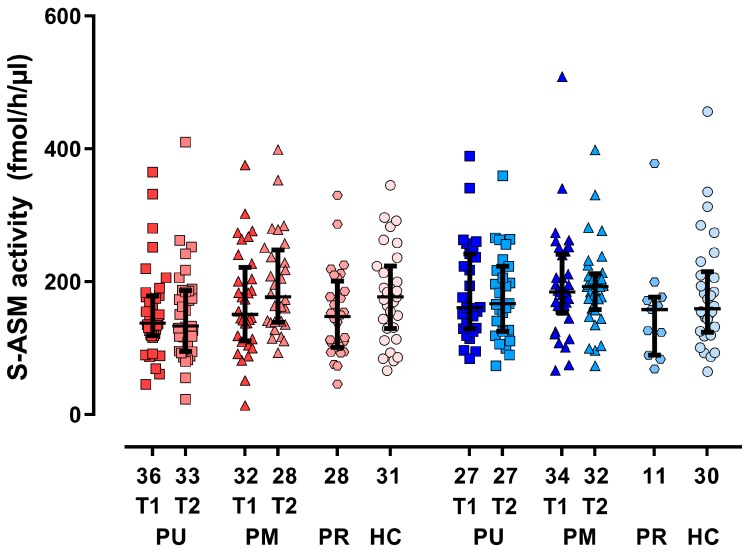
Serum acid sphingomyelinase (S-ASM) activity for female (red) and male (blue) subgroups of patients with a current major depressive episode (PU unmedicated, PM medicated, T1 at inclusion, T2 at follow-up approximately 3 weeks later), patients in remission (PR), and healthy controls (HC). Boxplots show individual data, the median and the interquartile range. The numbers of male and female individuals are provided below the x-axis.

The activity of S-ASM did not differ significantly between males and females of the whole group nor for patient groups and controls (Table 1). We, therefore, first performed tests on the mixed sample with subsequent exploratory sex-specific analyses. The S-ASM activities of patients with current MDE (*p* = 0.239 for unmedicated and *p* = 0.830 for medicated) and patients with remitted MDD (*p* = 0.067) did not significantly differ from the enzyme levels in healthy controls (Figure 1, Table 1). This was also true for sex-specific analyses. The median S-ASM activities were 13% lower in unmedicated patients and remitted patients compared to healthy subjects, whereas those of medicated patients were similar to controls. The enzyme activities between patients with and without medication differed nominally significantly only at follow-up in the mixed (*p* = 0.013) and female (*p* = 0.015) subgroups, but not in the male subgroup (*p* = 0.475).

Although inpatients were more severely depressed than outpatients, these groups did not differ in their S-ASM activities at inclusion (*p* = 0.182 for the total group of patients, *p* = 0.498 for unmedicated, *n* = 28 vs. 61; *p* = 0.851 for medicated patients, *n* = 35 vs. 5).

Furthermore, S-ASM activities did not decrease during the approximately 3 weeks of treatment as usual, but rather increased in medicated patients (10% increase, *p* = 0.047), but not in unmedicated patients (*p* = 0.825, Table 1). With 12%, the slight increase was most prominent in female initially medicated patients (*p* = 0.023). At follow-up, S-ASM activities of both patient groups with current MDE were still not different from healthy controls (*p* = 0.277 for unmedicated and *p* = 0.179 for medicated), also when analyzing sex-specific subsamples.

We subdivided individuals according to the classification of their (antidepressant or other) medication as FIASMA or not. There were no differences in S-ASM activities, not for the day of inclusion or at follow up, and no effect on the time course of ASM for the sex-mixed group. However, at inclusion, S-ASM levels were 14% (*p* = 0.022) higher in those males (combined groups due to low numbers) taking FIASMAs (*n* = 15) compared with those without (*n* = 87). Similarly, at follow-up, S-ASM levels were 22% (*p* = 0.045) higher in those males taking FIASMAs (*n* = 14) compared with those without (*n* = 44). For detailed documentation, we compared S-ASM activities of patients taking a specific drug (in total 91 different substances) or class of psychotropic drugs with levels in the absence of these drugs (Supplementary Table S3). There were only very weak effects, partially attributable to the small group sizes. Overall, S-ASM activity was lower in drug-free healthy controls compared to those individuals taking any drug (*p* = 0.006).

Concerning comorbidities, we observed low numbers of bipolar disorder, panic disorder and a number of cases with agoraphobia, social phobia, specific phobia, or generalized anxiety disorder in our cohort, however with no influence on S-ASM activities (Supplementary Table S4). In contrast, nicotine dependence exerted some effect—S-ASM activity was increased by 23% in smokers versus ex-smokers and non-smokers with similar levels (*p* = 0.011).

### 3.3. S-ASM Activity, Depression Severity, and Prospective Course

**Figure 2:**
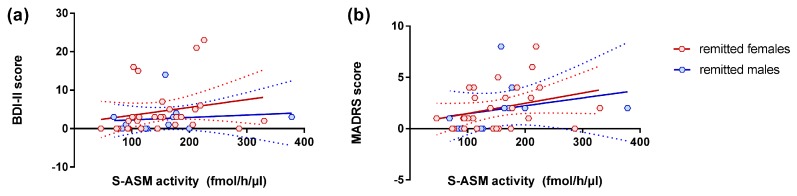
Positive correlation of serum acid sphingomyelinase (S-ASM) activity with depression severity assessed by (**a**) BDI-II (self-report) and (**b**) MADRS (administration by a clinician) inventories for female (red) and male (blue) remitted patients at study inclusion. Individual data with linear regression line and 95% confidence intervals.

We did not find a correlation between S-ASM activity and depression severity for any of the assessed scores (HAM-D, MADRS, and BDI-II) in the total or sex-specific cohorts (all *p* > 0.3). Such a relationship was also absent in the groups of medicated and unmedicated patients, as well as controls (all *p* > 0.08). However, in the subgroup of patients with remitted MDD, high S-ASM activities were related to high BDI-II and MADRS scores in the total group with comparable effect sizes in males and females (Table 2, Figure 2).

**Figure 3:**
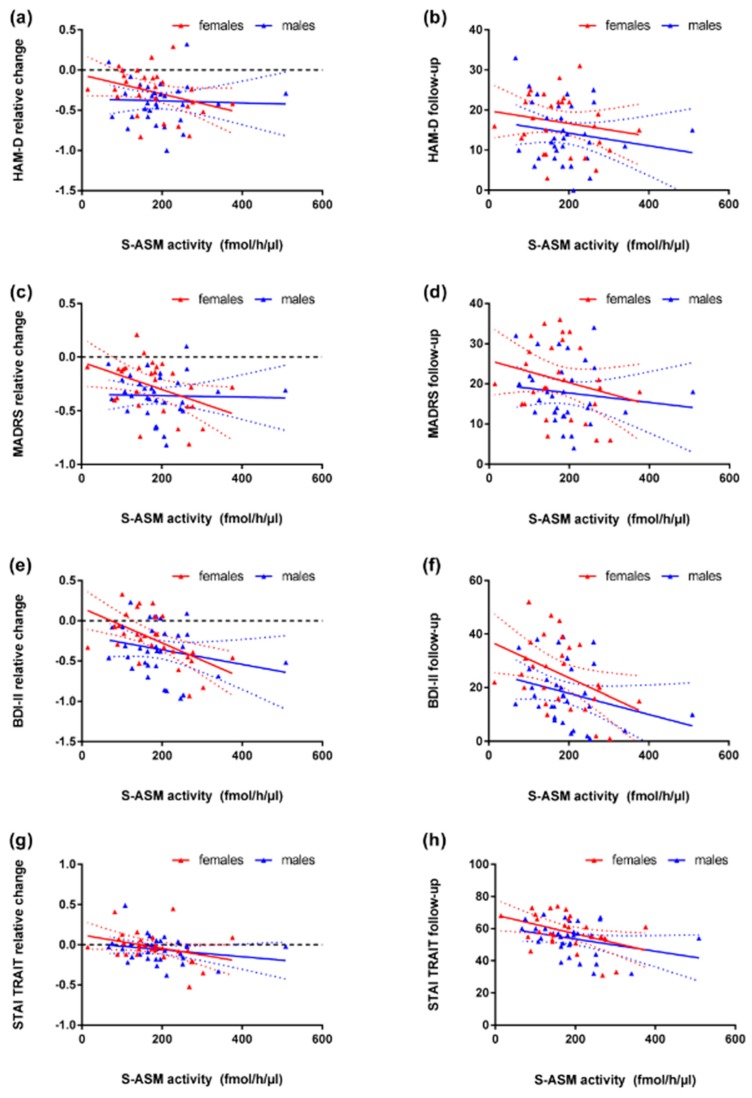
Correlation of serum acid sphingomyelinase (S-ASM) activity at inclusion with change in depression severity from inclusion to follow-up (approximately 3 weeks) and score at follow-up assessed by MADRS (**a**,**b**), HAM-D (**c**,**d**, both administered by a clinician), and BDI-II (**e**,**f**, self-report) inventories for female (red) and male (blue) medicated patients with current major depressive episode. High S-ASM activity is related to the stronger improvement of depression in the whole group and particularly the female subgroup. (**g**,**h**) Correlation of S-ASM activity at inclusion with the change of STAI trait score during treatment and with the score at follow-up in medicated patients. Individual data with linear regression line and 95% confidence intervals.

The analysis of the progression of depression revealed strong associations between high S-ASM activity at inclusion and a stronger improvement (all rho ≤ −0.300, all *p* < 0.020) and lower depression levels at follow-up (all rho < −0.200, all *p* ≤ 0.1) in the group of medicated depressed patients. This effect was observed for all three depression scales (Table 3) and driven by the female sub-cohort, whereas it was nearly absent in males (Figure 3a–f).

### 3.4. S-ASM Activity and State and Trait Anxiety (STAI)

In agreement with the high rate of comorbidity between depression and anxiety, sum scores for state and trait anxiety (theoretical range 20–80) were increased (by nearly two-fold) for medicated and unmedicated patients compared to healthy controls close to or above the suggested cut-off points for clinical significance [41]. These scores were only slightly elevated in remitted patients (Table 1). Therefore, similarly to depression severity scores, we analyzed the correlation of S-ASM activity with these anxiety scores.

While S-ASM activity did not show any significant association with the STAI trait sum score at inclusion (neither in the total cohort nor sex- or group-specific subsamples), it correlated strongly (both rho < −0.3) with the score at follow-up and with the change in score specifically in the group of medicated patients with current MDE (Table 3). High enzyme activity was related to lower trait anxiety and a stronger decrease in the sum score. This effect was mostly driven by female patients, but a similar trend was present in males (Figure 3g,h). Since benzodiazepines are used as anxiolytic drugs, this analysis was repeated for medicated patients taking this drug class (alprazolam, diazepam, flurazepam, lorazepam, *n* = 6 medicated patients with follow-up data). While STAI trait scores did not differ between these patients and those without benzodiazepines, the associations of S-ASM activity with the score at follow-up (rho = −0.841 and *p* = 0.036) and with the change in score (rho = −0.853 and *p* = 0.031) was even stronger than in the mixed group indicating a higher predictive potential despite the small group size.

Interestingly, the analysis at the individual item-level revealed a high correlation exclusively between S-ASM activity and improvement in the seven anxiety-absent items with reversed scoring weights (#1, #6, #7, #10, #13, #16, and #39; such as #10 “I am happy.” or #16 “I am content.”) with all rho > 0.4 (for raw scores prior to inversion) and all *p* < 0.02. The other 13 items concerning negative aspects of anxiety (such as #11 “I have disturbing thoughts.” or #12 “I lack self-confidence.”) of the STAI trait scale were not associated with S-ASM with all |rho| < 0.25 and all *p* > 0.18 (Supplementary Table S5).

For STAI state sum scores, there were no associations between initial S-ASM activity, neither in the total cohort nor sex- or group-specific subsamples. Only a weak association of S-ASM activity with the score at follow-up again for medicated patients (rho = −0.269, *p* = 0.037 for all, rho = −0.139, *p* = 0.481 for females and rho = −0.320, *p* = 0.075 for males) was observed. Similar to the STAI trait score, high enzyme activity was related to lower state anxiety at the follow-up visit but not at inclusion. However, we did not find a significant correlation with the relative change in the sum score.

### 3.5. S-ASM Activity and Self-Reported Health-Related Quality of Life (SF-12)

We assessed self-reported health-related quality of life using the SF-12 inventory and found that only medicated and unmedicated patients with current MDE, but not remitted patients, presented with lower scores than controls (Table 1). As expected, the reduction was considerably stronger for the mental than for the physical component score.

**Figure 4:**
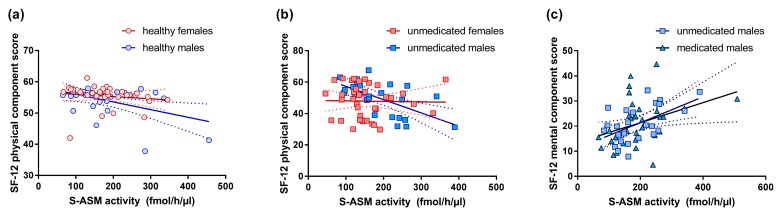
Correlation of serum acid sphingomyelinase (S-ASM) activity at inclusion with health-related quality of life assessed by the SF-12. High S-ASM levels were associated with a low physical component score in healthy individuals (**a**) and unmedicated patients with a current major depressive episode (**b**). High S-ASM levels were associated with a high mental component score in unmedicated and medicated male patients with a major depressive episode (**c**). Individual data with linear regression line and 95% confidence intervals.

Corresponding to the assumed association of peripheral S-ASM with inflammation or stress, the enzyme activities correlated negatively with the physical component score in healthy individuals (rho = −0.272, *p* = 0.035), particularly in females (rho = −0.422, *p* = 0.018, for males rho = −0.153, *p* = 0.427 despite the visual perception of a stronger effect in males from regression lines reflecting parametric testing), i.e., high S-ASM levels were associated with low physical quality of life such as body pain and low general health (Figure 4a). This effect was not present in remitted or medicated patients but was similarly observed in unmedicated depressed patients (rho = −0.289, *p* = 0.024). Here, the effect originated in the male subgroup (rho = −0.618, *p* = 0.001) and was not significant in females (Figure 4b).

Interestingly, the mental component score correlated with S-ASM activity in the opposite way—high enzyme levels were related to higher mental health, such as vitality, social functioning, and emotional role in depressed medicated patients (rho = 0.267, *p* = 0.033, Figure 4c), more specifically in medicated males (rho = 0.364, *p* = 0.034) but not females. This effect was even stronger in unmedicated male patients (rho = 0.492, *p* = 0.013) but not in female patients.

As implied by these observations, the physical component score correlated negatively with the mental component score in each group, but this reached nominal statistical significance only in unmedicated males (rho = −0.505, *p* = 0.010) and healthy females (rho = −0.526, *p* = 0.002).

### 3.6. S-ASM Activity and Serum Lipid Levels

A previous analysis of this cohort has revealed group differences for serum lipids and significant correlations between serum lipid markers and depression severity, as well as the prospective course of depression [32]. Further subdivision of the group of patients with current MDE shows nominally significantly higher values for triglycerides, total and LDL cholesterol, and HDL/LDL ratio, as well as lower HDL cholesterol in medicated patients, compared to healthy controls. However, none of these differences are significant in unmedicated patients with current MDE or patients with remitted MDD, although both groups show a similar weak trend (with the exception of HDL cholesterol) (Table 1).

**Figure 5:**
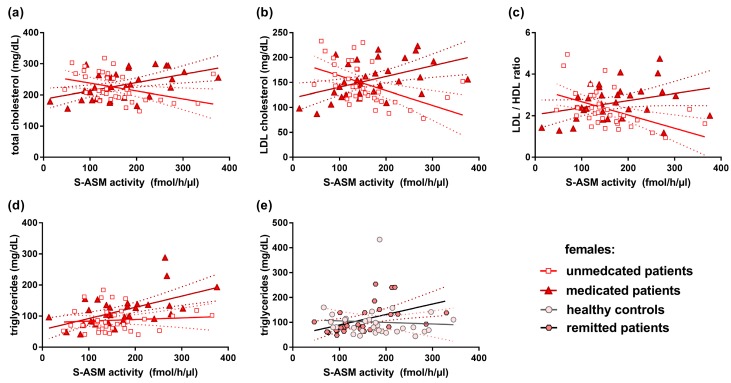
Correlation of serum acid sphingomyelinase (S-ASM) activity with serum lipids at the time of inclusion. High S-ASM levels were associated with total cholesterol (**a**), low-density lipoprotein (LDL) cholesterol (**b**), and ratio of low- to high-density lipoprotein cholesterol (LDL/HDL) (**c**) in unmedicated and medicated female patients and to total triglycerides in female medicated patients (**d**) and in female remitted patients (**e**). Individual data with linear regression line and 95% confidence intervals.

Interestingly, S-ASM activity was strongly and highly nominally significantly associated with these parameters in the females, but not in males, and with opposite direction for unmedicated versus medicated depressive patients (Table 4, Figure 5)—the higher total cholesterol, higher LDL cholesterol, and higher LDL/HDL ratio were associated with lower enzyme activities in unmedicated females but with higher activities in the medicated group. A positive correlation of S-ASM activity with triglycerides was only observed in medicated depressive females (rho = 0.533, *p* = 0.002) and in female remitted patients (rho = 0.626, *p* = 0.0003), similar to the previously reported positive association in healthy females [30].

### 3.7. Replication of S-ASM Correlation with Liver Enzyme Activities

We have previously demonstrated an association between serum S-ASM activity and the activities of the liver enzymes gamma-glutamyl transferase (GGT), alanine aminotransferase (ALT, also known as glutamic-pyruvic transaminase, GPT), and aspartate aminotransferase (AST, also known as glutamic-oxaloacetic transaminase, GOT), not only in alcohol-dependent patients, but for GGT and ALT (to a lesser extent) also in 200 healthy control subjects [30]. This positive correlation was replicated in the total cohort of 229 individuals (including depressed patients, with alcohol dependence as exclusion criterion) for GGT (rho = 0.188, *p* = 0.004), ALT (rho = 0.218, *p* = 0.0009), and AST (rho = 0.168, *p* = 0.011), with no interfering age-effect (rho = 0.047, *p* = 0.479), as opposed to the previous data. The strongest effects were present in the subgroup of 30 healthy male individuals (GGT, rho = 0.522, *p* = 0.003; ALT, rho = 0.414, *p* = 0.023; and AST, rho = 0.467, *p* = 0.009) with some potential influence of age (rho = 0.373, *p* = 0.043 for correlation with S-ASM) but was not present in the 31 healthy females (Supplementary Figure S1). Of note, there was no association of S-ASM activity with total serum protein or albumin levels neither in the whole cohort nor in sex- or group-specific subsamples.

## 4. Discussion

Based on the available animal model and human studies, we expected to find elevated levels of S-ASM activity in patients with a current MDE compared to patients with remitted MDD and healthy controls. However, such differences could not be identified in our clinical sample of moderately to severely affected patients, neither in unmedicated (antidepressant-free) nor medicated patients nor in sex-specific analyses, and also with no trend in the expected direction. The initial pilot study investigated 17 antidepressant-free patients of mixed sex with comparable severity of major depression based on HAM-D scores of 16–28, but analyzed the lysosomal enzyme in cultured peripheral blood mononuclear cells from these patients [16]. The secretory and lysosomal forms of ASM thus show different behavior concerning MDD in their general response to the physiological changes associated with the disease or their latency or extent of the response to such changes.

The absence of an increase is in contrast to the stronger response of S-ASM (3-fold higher levels than controls [28]) compared to L-ASM [27] to chronic alcohol consumption, suggesting that S-ASM could respond more sensitively to physiological alterations or at least to alcohol. The increased levels of plasma S-ASM (by 60%) in 11 combat veterans with post-traumatic stress disorder compared to five male controls [23] might be specific to this disease entity. This finding is not related to the difference in biological material since plasma and serum yield comparable enzyme activities [38]. Additional evidence for the role of sphingolipids in the pathogenesis of depression is mostly based on alterations of ceramide or sphingomyelin species in human blood [21,22] or animal tissues [24,25], suggesting an involvement of acid sphingomyelinase as an enzyme hydrolyzing sphingomyelin to ceramide. However, these changes could also be due to dysregulations of neutral sphingomyelinase, ceramidase species, or other enzymes of the catabolic and biosynthetic sphingolipid pathways. A recent study applying targeted and untargeted lipidomics for patients with MDD and bipolar disorder did not find an association of plasma ceramide species levels with the current episode, but strongly increased levels of ceramides and their hexosyl-metabolites in both patient groups, which were, however, not associated with improvement in MADRS scores [42].

Another finding was the absence of a difference in S-ASM activity between individuals taking FIASMA medication and those without. On the contrary, the sex-specific analysis revealed slightly increased levels in males under FIASMA treatment at inclusion, as well as during follow-up. Since FIASMAs have only been shown to inhibit the cellular lysosomal form of the enzyme in cell culture models [18] as well as in cultured peripheral blood mononuclear cells [16], by accumulating in the lysosome, displacing ASM from its interaction with the inner lysosomal membrane and thus exposing the protein to proteolytic degradation [18], they would not affect activity of the secreted form by the same mechanism. Contrariwise, FIASMAs could stimulate a feedback process with increased *SMPD1* gene expression to replace lost lysosomal ASM concomitant with additional production of the secretory form. However, future research is needed to validate this explanation. One very recent description of higher plasma ceramide levels in depressive patients receiving antidepressants (with unreported FIASMA status) compared to those without [42] supports a higher ASM activity under antidepressant therapy, but might also represent changes in other enzymes producing or degrading ceramide [11].

Consistent with the lack of increased enzyme activities in patients with current MDE, we also did not observe a decrease in S-ASM activity during approximately 3 weeks of treatment, during which depression severity scores decreased markedly.

Concerning the association of ASM activity with depression severity previously reported for the lysosomal enzyme [16] and a major criterion for the utility as a biomarker, we observed a positive correlation of S-ASM activities with BDI-II and MADRS scores, albeit exclusively in the subgroup of patients with remitted MDD and with scores in the range of no clinical depression. Contrary to our expectation, such a positive association of enzyme activity was not found in depressed patients for any of the assessed scores (HAM-D, MADRS, and BDI-II), neither in the total nor sex-specific cohorts (all *p* > 0.3).

To our knowledge, this is the first study to show a correlation between S-ASM activities at study inclusion and the prospective course of depression—high enzyme activities were related to both lower depression scores at follow-up (approximately 3 weeks later, for BDI-II) and a stronger improvement in all three depression scales including self-report and clinician assessment. However, this association was confined to the group of medicated patients taking antidepressants in a stable regime for at least 2 weeks and was absent in depressed patients free of antidepressants for at least 2 weeks. There are some differences between these groups of patients with current MDE that could contribute to this differentiation (Table 1). Medicated patients presented with a higher BMI (*p* = 0.0004), almost two-fold higher levels of the inflammatory marker C-reactive protein (CRP, *p* = 0.005)—both parameters were not associated with S-ASM in unmedicated or medicated patients. Initial S-ASM activities were slightly higher in medicated patients (*p* = 0.166) and increased to even higher levels (*p* = 0.013) compared to the unmedicated group. Medicated patients responded better to therapy during the approximately 3 weeks observation period, with significantly larger decreases of HAM-D and MADRS depression scores (both *p* = 0.010), which is consistent with their stronger improvement with higher initial S-ASM activity also for comparing both groups. It should be noted that initial depression severity was slightly higher in medicated patients particularly for HAM-D (*p* = 0.064) and MADRS (*p* = 0.057) scores, which could partially explain the stronger decrease in score. Moreover, we expect that the previously initiated medication also facilitates symptom reduction.

Finally, we analyzed the relationship between S-ASM and triglycerides and cholesterol species as these might influence lipid-metabolizing enzymes, including ASM. Unmedicated and medicated patients differed in several blood lipid parameters (higher values in medicated patients for triglycerides, total cholesterol, LDL/HDL ratio, all *p* < 0.007) with a strong and nominally highly significant association of these parameters with S-ASM in the opposite direction (see 2.6. and Figure 5). In view of the recent finding that high levels of these lipids (total cholesterol, LDL cholesterol, and LDL/HDL ratio) predict a stronger decline in HAM-D scores [32], S-ASM and lipids could interact in a mechanism leading to an amelioration in medicated patients that is absent or (perhaps) less active in unmedicated ones.

Replication of these results is certainly needed in patients of both sexes with sufficient group size to be able to differentiate between different therapeutic approaches (behavioral versus pharmacological using antidepressants with or without FIASMA activity) and possibly control for the influence of genetic effects, which have been found to be associated with the risk of MDD in individuals of European descent [43]. Even in case of success, S-ASM activity alone and in combination with serum lipids would certainly still need to be accompanied by additional markers for a diagnostic pattern of MDD, taking into account the complex and heterogeneous presentation of this illness [44]. New high-throughput screening technologies for the quantification of omics data combined with novel computational approaches, including machine intelligence and deep learning, are revolutionizing biomarker research to deliver multi-parametric disease signatures and identify functional networks [45].

Both clinical and epidemiological studies indicate a high comorbidity of depression and anxiety, with rates up to 40–80% [46,47], and there is strong evidence for similar etiological and maintenance processes underlying depressive and anxious psychopathology. Both disorders share many similar genetic [48], familial, and environmental risk factors [46,49], as well as behavioral aspects, causing difficulties in separating, for example, general anxiety disorder from MDD, even when applying neurobiological biomarkers in addition to clinical questionnaires [50]. There is evidence for a link between anxiety and ASM from a transgenic mouse model overexpressing ASM, where an impaired social preference and a depressive- and anxiogenic-like phenotype was observed in female, but not in male, animals, which was normalized by treatment with the antidepressant and FIASMA amitriptyline [51]. In humans, to our knowledge, only one study has analyzed the relationships between plasma phospho- and sphingolipid species and anxiety symptoms. In that study, both the absolute and relative amount of a specific phosphatidylcholine species were significantly related to anxiety symptoms of the Hospital Anxiety and Depression Scales in this Dutch family-based study [22]. We assessed state and trait anxiety using the STAI and observed that high S-ASM activity coincided with a lower score at follow-up, as well as with a larger change in score specifically in the group of medicated patients with current MDE – associations similarly identified for the depression severity scores. Surprisingly, this association was found for more stable personality characteristic trait anxiety (as opposed to state anxiety—a temporary feeling with similar weaker or absent correlations with S-ASM). However, the sub-item analysis revealed that only anxiety-absent items of the trait questionnaire contributed to this correlation, whereas negative aspects of trait anxiety were not related to S-ASM. This distinction highlights that the correlation of S-ASM with the STAI trait score seems to represent a relation to the amelioration of mood (i.e., a stronger decline and less depression severity at follow-up for medicated patients with higher enzyme levels). It also supports the above-reported associations of S-ASM and depression scores.

Elevated S-ASM activities in some studies, paralleled by an accumulation of ceramide, have been found to be associated with various pathophysiological conditions in humans, not only for psychiatric disorders but also heart disease [52] or non-alcoholic fatty liver disease and a number of severe inflammatory diseases, such as hepatitis C, sepsis, or inflammatory renal disease [15]. An association between high levels of S-ASM (thought to result from the enhanced secretion and activation of the enzyme by oxidative stress) and cytokines with a lower physical component score assessed by the SF-12 quality of health questionnaire is, thus, not surprising. Similar to the perturbations of mitochondrial function and homeostasis in ASM deficiency [53], the hyperactivity of ASM could lead to the transcriptional repression of mitochondrial biogenesis and, via a disturbed energy metabolism, to compromised physical health. However, this indirect relationship was only observed in medication-free individuals (unmedicated patients and healthy controls), although scores of medicated patients did not differ from those of unmedicated patients and scores of remitted patients did not differ from controls. Presumably, antidepressant drugs modified this association through alteration of (perceived) physical well-being or of S-ASM activity uncoupling these parameters. An additional factor influencing the physical quality of health is the classical and reliable marker of chronic inflammation, CRP, which was also found to be related to a poor physical health score in non-psychiatric populations [54], as well as in patients with depression [55], and was increased in our medicated patients (Table 1). Taking into account confounding factors similar to this publication using the long SF-36 questionnaire and available in our study (sex, age, education years, depression severity, STAI state, and CRP) in a linear regression model, the relationship between high S-ASM activity and impaired physical health remained nominally significant in the groups of unmedicated patients (*p* = 0.020) and healthy controls (*p* = 0.037) and was not present in medicated depressive patients and in remitted patients.

Concerning the SF-12 mental component score, we observed the opposite relationship to the physical component score exclusively in male patients with current MDE. High S-ASM levels were related to higher mental health, and this could relate to the observed better prospective course of depressed patients with high S-ASM levels via mechanisms of better coping and resilience.

These data point to an unexpected inverse association between physical and mental component scores, which was indeed detected in all groups, most strongly in healthy females and unmedicated males. While surprising, some indication of this effect—or at least the lack of a highly positive relationship—has previously been reported [56], with a Pearson’s correlation of *r* = −0.08 between SF-12 sum scores in adults with a mental health condition and *r* = 0.05 for the US general population. A comparison of correlations between sum scores and subscales revealed the lowest associations between the physical component sum and the emotional role or mental health and between the mental component sum and physical functioning (all *r* < 0.2).

The present study should also be interpreted with the following limitations in mind. Although our cohort was mostly sex-balanced and sufficiently large to identify relevant effects, including sex-specific or group-specific associations, the detection of smaller effects and subdivision, for example, according to treatment, would require a considerably larger sample size. Our natural population recruited at a university hospital institution and an academic surrounding might not be representative of patients and healthy controls in other regions or cultures. We did not correct the detected correlations for cofactors such as age, BMI, and educational background since exploratory analysis revealed a minor influence with the identified effects remaining observable. Additional potential confounding factors, such as employment, physical activity, diet, or sleep have not been taken into account. We admit that due to the high number of exploratory tests beyond the primary hypotheses, many *p* values would not survive correction for multiple testing. Clearly, our associational study design does not allow us to draw causative conclusions or to assume direct physiological links with S-ASM activity, although the predictive potential of S-ASM at inclusion on the course of depression includes directionality. On the other hand, the differentiation of patients into those with and without prior medication, as well as the inclusion of patients with remitted MDD, are specific strengths of our study. Even though the follow-up period of approximately 3 weeks was relatively short and a longer time frame to identify responders or relapse would be interesting, the second visit still allowed us to analyze and to identify the predictive potential of S-ASM activity.

It would be interesting to analyze how further factors associated with ASM activity, such as polymorphisms [15], transcriptional and post-translational modifications [13], and splice variants of *SMPD1* [57], reported to influence enzyme activity and to be altered in major depression [58], are influencing the observed correlations. The activity of S-ASM in cerebrospinal fluid [59], which could be determined in neurological patients with comorbid depression, would be a closer indicator of cerebral processes. Furthermore, quantification of the activity of other sphingolipid enzymes and serum ceramides and sphingomyelin species by mass spectrometry might help to explain the association of sphingolipids and depression.

## 5. Conclusions

Based on previous data with increased lysosomal ASM in patients with major depression, which correlated with depression severity, and the lack of this effect in our moderately to severely affected patients with the current major depressive episode for S-ASM, we conclude that secretory and lysosomal isoforms of ASM behave differently in the context of major depression. Although S-ASM could not serve as a diagnostic marker for a current major depressive episode, we identified an association of high S-ASM activity with a stronger improvement of depression assessed independently by self-report and clinician’s interview in medicated patients. S-ASM could thus be developed as a useful biomarker for predicting the course of depression apparently only after stabilization of medication but not in initially unmedicated patients.

Our data with sex-specific effects emphasize the importance of separate analysis of female and male subgroups. We have also discovered novel associations of S-ASM with anxiety and physical as well as mental qualify of life, which warrant further investigations, and we replicated relationships of S-ASM with serum lipids and liver enzyme activities to consolidate previous findings.

Research on the involvement of ASM and related sphingolipid metabolizing enzymes in depression should be continued with larger cohorts of both sexes and include a longitudinal setting over a larger time window, also considering patients with remitted MDD to differentiate between state and trait markers of depression. Our study should be considered as an addition to the broad perspective of exploring mechanisms underlying the involvement sphingolipid metabolic pathways and traffic in psychiatric diseases to identify diagnostic and prognostic biomarkers and to develop novel possibly individualized therapeutic approaches.

## Figures and Tables

**Table 1:** Study cohort description and group differences for patients and control subjects at study inclusion and follow-up (3 weeks later).

Parameters	PU	PM	PR	HC	*p* Values for Group Difference				*p* Values for Sex Difference			
					PU vs. PM	PU vs. HC	PM vs. HC	PR vs. HC	PU	PM	PR	HC
*n* (females/males) at inclusion	36/27	32/34	28/11	31/30	0.325	0.480	0.793	**0.038**				
*n* (females/males) at follow-up	34^1^/27^2^	28/32	-/-	-/-	0.318							
age (years)	47 (34–53)	46 (33–54)	49 (46–58)	42 (32–54)	0.908	0.455	0.609	**0.019**	0.692	0.603	0.132	0.168
total education years ^a^	15 (13–18)	14 (13–16)	14 (13–16)	15 (13–18)	0.062	0.933	0.080	0.221	0.172	0.354	0.225	0.087
BMI (kg/m²)	25.3 (22.5–27.8)	28.5 (24.4–30.4)	25.7 (23.0–29.1)	24.4 (23–27.7)	**<0.001**	0.760	**0.001**	0.281	0.090	0.182	0.528	0.279
BDI-II score at inclusion	28 (22–34)	29 (24–35)	3 (0–4)	1 (0–3)	0.432	**<0.001**	**<0.001**	0.280	0.416	0.020	0.414	0.683
BDI-II score at follow-up ^c^	20 (15–25)	20 (13–31)			0.939				0.154	**0.041**		
BDI-II score at relative change ^c^	−0.27 (−0.42–−0.12)	−0.32 (−0.51–−0.07)			0.508				0.161	0.134		
HAM-D score at inclusion	21 (19–24)	23 (20–26)	2 (0–3)	1 (0–2)	0.064	**<0.001**	**<0.001**	**0.018**	0.818	0.151	0.678	**0.050**
HAMD-D score at follow-up ^c^	18 (14–21)	15 (10–22)			0.189				0.529	0.150		
HAMD-D score at relative change ^c^	−0.15 (−0.38–−0.05)	−0.32 (−0.51–−0.16)			**0.010**				0.788	0.083		
MADRS score at inclusion	26 (23–28)	28 (24– 34)	1 (0–3)	0 (0–2)	0.057	**<0.001**	**<0.001**	**0.012**	0.460	0.322	0.818	0.069
MADRS score at follow-up ^c^	21 (18–25)	18 (13–26)			0.143				0.398	0.170		
MADRS score relative change ^c^	−0.19 (−0.34–−0.07)	−0.32 (−0.46–−0.14)			**0.010**				0.387	0.094		
STAI state score at inclusion	50 (40–57)	54 (43–63)	32 (26–36)	28 (26–31)	0.080	**<0.001**	**<0.001**	**0.036**	0.160	0.142	0.158	0.238
STAI state score at follow-up ^c^	46 (37–52)	47 (42–57)			0.261				0.210	0.157		
STAI state score relative change ^c^	−0.06 (−0.15–0.02)	−0.05 (−0.14–0.09)			0.583				0.754	0.917		
STAI trait score at inclusion	62 (56–67)	61 (52–67)	33 (26–40)	28 (25–33)	0.261	**<0.001**	**<0.001**	**0.007**	0.486	0.261	0.116	0.448
STAI trait score at follow-up ^c^	59 (54–63)	56 (51–65)			0.530				0.911	0.121		
STAI trait score relative change ^c^	−0.06 (−0.12–0.01)	−0.04 (−0.14–0.03)			0.558				0.703	0.142		
SF-12 physical component score ^b^	50.9 (38.7–57.6)	50.9 (43.8–57.1)	55.5 (50.1–56.5)	55.7 (54.9–56.7)	0.432	**0.001**	**0.003**	0.124	0.524	0.206	0.390	0.097
SF-12 mental component score ^b^	19.9 (16.6–27.6)	18.5 (14.3–26.6)	53.2 (48.7–57.8)	56.2 (52.1–58.9)	0.165	**<0.001**	**<0.001**	0.240	0.486	0.501	0.140	0.351
CRP (mg/L)	0.9 (0.5–2.1)	1.6 (1.1–3.1)	1.4 (0.9–2.2)	1.3 (0.7–2.3)	**0.005**	0.139	0.127	0.373	0.813	0.913	0.221	0.129
Triglycerides (mg/dL)	90 (68–124)	127 (97–174)	93 (68–138)	81 (63–142)	**<0.001**	0.944	**<0.001**	0.445	**0.003**	**0.003**	0.939	0.665
Total cholesterol (mg/dL)	217 (187–264)	235 (198–267)	227 (201–251)	208 (181–248)	0.256	0.313	**0.028**	0.198	0.868	0.719	0.083	0.226
HDL cholesterol (mg/dL)	59 (52–66)	51 (43– 64)	58 (46–68)	58 (50–69)	**0.007**	0.976	**0.018**	0.641	**<0.001**	**<0.001**	**<0.001**	**<0.001**
LDL cholesterol (mg/dL)	143 (118–179)	159 (132–196)	151 (139–167)	134 (117–167)	**0.070**	0.182	**0.001**	0.057	0.433	0.151	0.414	0.608
HDL/LDL ratio	2.4 (1.9–3.3)	3 (2.3–4)	2.6 (1.9–3.3)	2.2 (1.8–3.0)	**0.003**	0.270	**<0.001**	0.158	**0.002**	**<0.001**	**0.037**	0.055
GGT (U/L)	21 (15–30)	25 (17–42)	18 (13–32)	19 (15–26)	**0.044**	0.527	**0.012**	0.966	**0.001**	**<0.001**	0.167	0.104
ALT (U/L)	19 (15–29)	26 (17–41)	18 (15–26)	22 (16–31)	**0.043**	0.444	0.209	0.210	**<0.001**	**<0.001**	0.040	**0.002**
AST (U/L)	23 (19–26)	24 (21–32)	24 (20–28)	26 (22–30)	0.118	**0.005**	0.250	0.072	**0.033**	**0.001**	0.158	**0.002**
S-ASM (fmol/h/µL serum) at inclusion	151 (121–206)	176 (125–228)	150 (101–186)	173 (130–214)	0.166	0.237	0.828	0.068	0.080	0.191	0.939	0.863
S-ASM (fmol/h/µL serum) at follow-up ^c^	160 (114–202)	189 (143–227)			**0.013**				0.076	0.917		
S-ASM relative change ^c^	0.03 (−0.19–0.22)	0.10 (−0.08–0.28)			0.200				0.994	0.273		

The table shows frequencies and median with interquartile range and *p* values from Mann-Whitney-U tests comparing patient groups with healthy control subjects or females with males. See Supplementary Tables S1 and S2 for sex-specific data. Missing values—a < 13%, b < 3%, and c < 1%. Mann–Whitney U-test or χ^2^ test with nominal *p* < 0.05 in bold. Groups—PU unmedicated depressive patients, PM medicated depressive patients, HC healthy controls, and PR patients with remitted major depressive disorder; Parameters—BMI body mass index, BDI-II Beck Depression Inventory-II, HAM-D Hamilton Depression Rating Scale, MADRS Montgomery–Åsberg Depression Rating Scale, STAI State-Trait Anxiety Inventory, SF-12 self-reported health-related quality of life, CRP C-reactive protein, HDL high-density lipoprotein, LDL low-density lipoprotein, GGT gamma-glutamyl transferase, ALT alanine aminotransferase (glutamic-pyruvic transaminase, GPT), AST aspartate aminotransferase (glutamic-oxaloacetic transaminase, GOT), and S-ASM secretory acid sphingomyelinase. ^1^ One of the 34 females provided only psychometric data but no blood at the follow-up visit. ^2^ Psychometric data are missing from one male patient (out of 27) who provided blood at the beginning of the follow-up visit.

**Table 2:** Positive correlation of serum acid sphingomyelinase (S-ASM) activity with depression severity scores at inclusion as assessed by rating by a clinician (HAM-D and MADRS) and self-rating (BDI-II) in patients with remitted major depressive disorder.

S-ASM Activity		HAM-D		MADRS		BDI-II	
	*n*	rho	*p*	rho	*p*	rho	*p*
All	39	0.143	0.386	**0.451**	**0.004**	**0.385**	**0.015**
Female	28	0.146	0.459	0.368	0.054	**0.424**	**0.024**
Male	11	0.155	0.648	**0.667**	**0.025**	0.259	0.442

Rho and *p* values from Spearman correlations, nominal *p* < 0.05 in bold.

**Table 3:** Serum acid sphingomyelinase (S-ASM) activity at inclusion predicts relative change of depression severity and score at follow-up (approximately 3 weeks post inclusion) as assessed by rating by a clinician (HAM-D and MADRS) and self-rating (BDI-II), as well as relative change of the STAI-TRAIT score and score at follow-up in patients with a current major depressive episode (MDE).

S-ASM Activity			Relative Change of Score from Inclusion to Follow-Up								Sum Score at Follow-Up							
			HAM-D		MADRS		BDI-II		STAI (Trait)		HAM-D		MADRS		BDI-II		STAI (Trait)	
		*n*	rho	*p*	rho	*p*	rho	*p*	rho	*p*	rho	*p*	rho	*p*	rho	*p*	rho	*p*
Unmedicated	all	60	0.231	0.075	0.066	0.618	−0.112	0.396	−0.149	0.256	0.082	0.534	−0.054	0.682	−0.007	0.956	0.044	0.736
Patients	female	34	0.136	0.443	−0.024	0.895	−0.148	0.403	−0.127	0.476	0.021	0.904	−0.100	0.575	0.059	0.741	0.265	0.129
With current MDE	male	26	0.295	0.144	0.236	0.245	0.069	0.736	−0.119	0.563	0.213	0.295	0.100	0.628	−0.123	0.551	−0.269	0.184
Medicated	all	60	**−0.300**	**0.020**	**−0.306**	**0.017**	**−0.411**	**0.001**	**−0.344**	**0.007**	-0.206	0.114	−0.240	0.065	**−0.367**	**0.004**	**−0.376**	**0.003**
Patients	female	28	**−0.400**	**0.035**	**−0.401**	**0.035**	**−0.585**	**0.001**	**−0.406**	**0.032**	-0.182	0.354	−0.202	0.302	**−0.389**	**0.041**	**−0.395**	**0.037**
With current MDE	male	32	−0.094	0.607	−0.130	0.479	−0.184	0.314	−0.231	0.203	-0.127	0.487	−0.158	0.388	−0.271	0.134	−0.291	0.106

Rho and *p* values from Spearman correlations, nominal *p* < 0.05 in bold. HDL, high-density lipoprotein; LDL, low-density lipoprotein.

**Table 4:** Opposite correlation of serum acid sphingomyelinase (S-ASM) activity with serum lipids in female unmedicated and medicated depressive patients with a current major depressive episode.

S-ASM Activity		Triglycerides		Total Cholesterol		LDL Cholesterol		LDL/HDL Ratio	
	*n*	rho	*p*	rho	*p*	rho	*p*	rho	*p*
Unmedicated females	36	0.103	0.549	**−0.531**	**0.0009**	**−0.596**	**0.0001**	**−0.571**	**0.0003**
Medicated females	32	**0.533**	**0.002**	**0.481**	**0.005**	**0.487**	**0.005**	0.302	0.093

Rho and *p* values from Spearman correlations, nominal *p* < 0.05 in bold.

## Supplementary Materials

Supplementary materials can be found at https://www.mdpi.com/2077-0383/8/6/846/s1. Figure S1: Positive correlation of serum acid sphingomyelinase (S-ASM) with liver enzyme activities GGT, ALT, and AST in the total cohort (*n* = 229, a–c) and the subgroup of male (*n* = 30) but not female (*n* = 31) healthy controls (d–f). GGT gamma-glutamyl transferase, ALT alanine aminotransferase (glutamic-pyruvic transaminase, GPT), AST aspartate aminotransferase (glutamic-oxaloacetic transaminase, GOT). Table S1: Sex-specific demographic and laboratory data for females corresponding to Table 1 for the whole groups. See the legend of Table 1 for details. Table S2: Sex-specific demographic and laboratory data for males corresponding to Table 1 for the whole groups. See the legend of Table 1 for details. Table S3: Comparison of activities of secretory acid sphingomyelinase (S-ASM) in the serum of patients and controls depending on specific medications. Table S4: Comparison of activities of secretory acid sphingomyelinase (S-ASM) in the serum of patients and controls depending on comorbidities and smoking status. Table S5: Correlation between serum acid sphingomyelinase (S-ASM) activity and change of the 20 sub-item scores of the STAI trait inventory between inclusion and follow-up (approximately 3 weeks) in medicated female depressed patients (*n* = 32, rho and *p* values from Spearman correlations, nominal *p* < 0.05 in bold).

## Abbreviations

ALT	Alanine aminotransferase (glutamic-pyruvic transaminase, GPT)
ASM	Acid sphingomyelinase
AST	Aspartate aminotransferase (glutamic-oxaloacetic transaminase, GOT)
BDI-II	Beck Depression Inventory-II
BMI	Body mass index
GGT	Gamma-glutamyl transferase
FIASMA	Functional inhibitor of acid sphingomyelinase
HAM-D	Hamilton Depression Rating Scale
HDL	High-density lipoprotein
L-ASM	Lysosomal acid sphingomyelinase
LDL	Low-density lipoprotein
MADRS	Montgomery–Åsberg Depression Rating Scale
MDE	Major depressive episode
MDD	Major depressive disorder
CRP	C-reactive protein
S-ASM	Secretory acid sphingomyelinase
SF-12	Self-reported health-related quality of life
*SMPD1*	Sphingomyelin phosphodiesterase 1 gene encoding ASM
STAI	State-Trait Anxiety Inventory
