# Supplementary material for: Secretory Acid Sphingomyelinase in the Serum of Medicated Patients Predicts the Prospective Course of Depression
Source: J Clin Med. 2019 Jun 13;8(6):846. doi: 10.3390/jcm8060846 (PMC6617165; doi:10.3390/jcm8060846)
Supplement: Supplementary file 1 [file jcm-08-00846-s001.pdf]

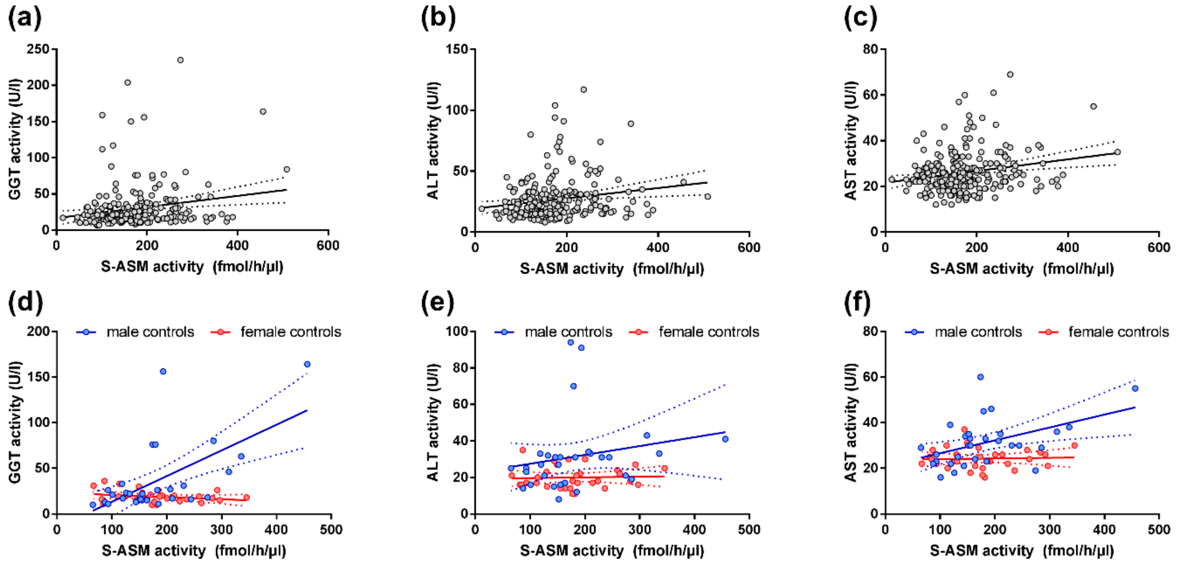

Supplemental Figure S1. Positive correlation of serum acid sphingomyelinase (S-ASM) with liver enzyme activities GGT, ALT, and AST in the total cohort (n = 229, **a**-**c**) and the subgroup of male (n = 30) but not female (n = 31) healthy controls (**d**–**f**). GGT gamma-glutamyl transferase, ALT alanine aminotransferase (glutamic-pyruvic transaminase, GPT), AST aspartate aminotransferase (glutamic-oxaloacetic transaminase, GOT).

Supplemental table S1. Sex-specific demographic and laboratory data for females corresponding to Table 1 for the whole groups. See legend of Table 1 for details.

Parameters					*p* values for group difference			
	**PU**	PM	PR	HC	PU vs. PM	PU vs. HC	PM vs. HC	PR vs. HC
n at inclusion	36	32	31	28				
n at follow-up	34	28						
age (years)	45 (33–53)	46 (32–55.5)	52 (47–63)	47 (32–60)	0.810	0.642	0.645	0.109
total education years [a]	15 (13–17)	14 (12–17)	14 (12–15)	14 (12–17)	0.216	0.527	0.585	0.792
BMI (kg/m²)	24.0 (21.3–27.3)	27.3 (22.1–30.6)	25.3 (22.7–29.2)	24.3 (23–26.2)	0.055	0.664	0.124	0.309
BDI-II score at inclusion	29 (21–36)	33 (27–39)	3 (0–5)	1 (0–4)	0.176	**<0.001**	**<0.001**	0.368
BDI-II score at follow-up [c]	22 (15–27)	24 (16–36)			0.318			
BDI-II score at relative change [c]	−0.23 (−0.42–−0.06)	−0.21 (−0.42–−0.01)			0.955			
HAM-D score at inclusion	22 (19–26)	24 (21–27)	2 (1–4)	1 (0–3)	0.095	**<0.001**	**<0.001**	0.256
HAMD-D score at follow-up [c]	18 (14–21)	17 (11–22)			0.804			
HAMD-D score at relative change [c]	−0.24 (−0.38–−0.05)	−0.24 (−0.44–−0.08)			0.471			
MADRS score at inclusion	26 (22–28)	28 (25–35)	1 (0–4)	1 (0–2)	**0.046**	**<0.001**	**<0.001**	0.216
MADRS score at follow-up [c]	21 (18–25)	20 (15–29)			0.755			
MADRS score relative change [c]	−0.18 (−0.30–−0.04)	−0.18 (−0.46–−0.10)			0.258			
STAI state score at inclusion	47 (40–56)	55 (48–66)	32 (30–35)	29 (26–32)	**0.009**	**<0.001**	**<0.001**	**0.034**
STAI state score at follow-up [c]	46 (36 – 51)	49 (45–59)			**0.047**			
STAI state score relative change [c]	−0.05 (−0.15–0.03)	−0.02 (−0.17–0.09)			0.794			
STAI trait score at inclusion	62 (56–68)	65 (53–67)	36 (28–41)	29 (25–35)	0.830	**<0.001**	**<0.001**	**0.013**
STAI trait score at follow-up [c]	58 (52–62)	58 (53–67)			0.666			
STAI trait score relative change [c]	−0.06 (−0.13–0.02)	−0.04 (−0.12–0.07)			0.497			
SF-12 physical component score [b]	49.6 (37.3–56.8)	50.6 (42.3–55.0)	54.3 (49.2–56.7)	56.3 (55.2–57.5)	0.709	**0.003**	**0.001**	**0.035**
SF-12 mental component score [b]	19.4 (16.6–29.9)	16.5 (12.0–26.9)	52.5 (48.1–57.0)	54.3 (50.8–58.2)	0.094	**<0.001**	**<0.001**	0.185
CRP (mg/L)	1.0 (0.6–2.3)	1.8 (0.90–3.4)	1.5 (1.0–2.2)	1.4 (0.8–3.3)	0.057	0.074	0.853	0.627
Triglycerides (mg/dL)	75 (57–108)	106 (84–136)	88 (69–139)	80 (64–111)	**0.003**	0.396	0.039	0.320
Total cholesterol (mg/dL)	211 (185–268)	226 (189–265)	237 (209–263)	217 (185–255)	0.511	0.970	0.449	0.335
HDL cholesterol (mg/dL)	65 (58–71)	59 (51–71)	62 (55–74)	66 (58–73)	0.180	0.720	0.141	0.366
LDL cholesterol (mg/dL)	140 (118–182)	152 (128–190)	154 (136–177)	134 (112–167)	0.258	0.763	0.167	0.154
HDL/LDL ratio	2.1 (1.7–2.8)	2.6 (1.9–3.1)	2.3 (1.9–3.1)	2 (1.7–2.5)	0.117	0.660	0.037	0.120
GGT (U/L)	18 (13–23)	18 (12–26)	18 (13–24)	18 (14–21)	0.863	0.965	0.896	1.000
ALT (U/L)	17 (14–23)	17 (13–24)	17 (14–23)	18 (16–24)	0.975	0.236	0.295	0.429
AST (U/L)	22 (19–25)	22 (19–25)	23 (19–27)	24 (21–27)	0.693	**0.038**	0.088	0.403
S-ASM (fmol/h/μL serum) at inclusion	138 (119–178)	151 (112–216)	148 (102–196)	177 (130–224)	0.363	0.090	0.573	0.133
S-ASM (fmol/h/μL serum) at follow-up [c]	133 (96–186)	178 (140–245)			**0.015**			
S-ASM relative change [c]	0.05 (−0.21–0.21)	0.12 (−0.01–0.28)			0.165			

Supplemental table S2. Sex-specific demographic and laboratory data for males corresponding to Table 1 for the whole groups. See legend of Table 1 for details.

					p **values for group difference**			
**Parameters**	**PU**	**PM**	**PR**	**HC**	**PU vs. PM**	**PU vs. HC**	**PM vs. HC**	**PR vs. HC**
n at inclusion	27	34	30	11				
n at follow-up	27	32						
age (years)	49 (35–53)	46 (33–53)	49 (33–53)	36.5 (30–49)	0.642	0.143	0.244	0.407
total education years [a]	16 (13–19)	14 (13–16)	15 (13–17)	16.5 (14–18)	0.073	0.936	**0.028**	0.413
BMI (kg/m^2)	25.7 (23.3–28.3)	28.5 (26.7–30.2)	25.8 (25.6–27.0)	25.0 (22.9–28.4)	**0.003**	0.854	**0.003**	0.424
BDI-II score at inclusion	28 (23–32)	27 (21–32)	1 (0–3)	2 (0–3)	0.810	**<0.001**	**<0.001**	0.851
BDI-II score at follow-up [c]	18 (15–22)	17 (10–27)			0.402			
BDI-II score at relative change [c]	−0.34 (−0.41–−0.15)	−0.36 (−0.56–−0.10)			0.448			
HAM-D score at inclusion	21 (19–23)	22 (20–25)	2 (0–3)	0 (0–1)	0.439	**<0.001**	**<0.001**	0.116
HAMD-D score at follow-up [c]	18 (14–20)	13 (9–21)			0.173			
HAMD-D score at relative change [c]	−0.13 (−0.38–−0.05)	−0.38 (−0.58–−0.24)			0.009			
MADRS score at inclusion	27 (24–29)	27 (23–34)	2 (0–2)	0 (0–1)	0.541	**<0.001**	**<0.001**	0.080
MADRS score at follow-up [c]	20 (18–24)	17 (13–23)			0.127			
MADRS score relative change [c]	−0.21 (−0.34–−0.09)	−0.34 (−0.49–−0.23)			**0.032**			
STAI state score at inclusion	52 (46–58)	53 (40–62)	26 (22–36)	27 (25–31)	0.839	**<0.001**	**<0.001**	0.965
STAI state score at follow-up [c]	48 (42–54)	45 (39–57)			0.639			
STAI state score relative change [c]	−0.08 (−0.14–0.02)	−0.05 (−0.12–0.07)			0.527			
STAI trait score at inclusion	62 (57–67)	58 (52–67)	31 (25–35)	27 (25–32)	0.085	**<0.001**	**<0.001**	0.591
STAI trait score at follow-up [c]	59 (54–64)	56 (50 - 60)			0.216			
STAI trait score relative change [c]	−0.06 (-0.11–−0.02)	−0.04 (−0.17–0.01)			0.701			
SF-12 physical component score [b]	51.6 (39.1–57.6)	52.2 (45.2–58.4)	56.2 (50.2–56.5)	55.7 (53.8–56.5)	0.529	0.143	0.416	0.835
SF-12 mental component score [b]	20.0 (16.6–26.3)	20.1 (15.4–26.0)	57.2 (50.8–58.9)	56.9 (52.6–58.9)	0.866	**<0.001**	**<0.001**	0.788
CRP (mg/L)	0.9 (0.5–2.1)	1.6 (1.1–2.7)	1.0 (0.8–2.4)	1.2 (0.6–1.9)	0.058	0.761	**0.044**	0.873
Triglycerides (mg/dL)	108 (79–134)	157 (112–191)	101 (65–127)	90 (60–175)	**0.003**	0.406	**0.010**	0.919
Total cholesterol (mg/dL)	224 (189–254)	244 (199–272)	211 (196–235)	198 (172–243)	0.299	0.185	**0.019**	0.828
HDL cholesterol (mg/dL)	52 (46–59)	45 (41–52)	45 (42–53)	51 (47–61)	**0.016**	0.879	**0.030**	0.080
LDL cholesterol (mg/dL)	152 (121–179)	173 (138–197)	148 (139–166)	131 (117–169)	**0.144**	0.123	**0.001**	0.315
HDL/LDL ratio	2.9 (2.3–3.7)	3.7 (2.8–4.5)	3.2 (2.5–3.7)	2.6 (2.0–3.3)	**0.007**	0.133	**<0.001**	0.174
GGT (U/L)	24 (18–43)	39 (23–53)	25 (17–32)	22 (15–33)	**0.023**	0.263	**0.003**	0.761
ALT (U/L)	26 (19–36)	33 (27–50)	23 (19–33)	29 (19–33)	**0.020**	0.955	**0.032**	0.942
AST (U/L)	25 (21–34)	28 (22–35)	27 (20–37)	30 (23–35)	0.227	0.128	0.471	0.344
S-ASM (fmol/h/μL serum) at inclusion	161 (129–242)	185 (157–240)	158 (90–177)	160 (126–210)	0.504	0.774	0.294	0.359
S-ASM (fmol/h/μL serum) at follow-up [c]	167 (126–223)	193 (159–211)			0.475			
S-ASM relative change [c]	0.00 (−0.17–0.28)	0.04 (−0.14–0.24)			0.704			

Supplemental table S3. Comparison of activities of secretory acid sphingomyelinase (S-ASM) in the serum of patients and controls depending on specific medications. Except for the first three lines, activities and the number of individuals (*n*) is provided for those taking the indicated drug. Mann–Whitney U-test for the comparison of activities with drug versus without drug with nominal $p < 0.05$ in bold. Groups: PU unmedicated depressive patients, PM medicated depressive patients, HC healthy controls, PR patients with remitted major depressive disorder; FIASMA - functional inhibitor of acid sphingomyelinase (less than 50% residual activity in a cell culture modell). Please note, that these drugs have been tested at higher concentrations than present in patients' blood. Class refers to the classification of psychotropic substances as indicated in the first lines. Reference: Kornhuber, J.; Tripal, P.; Gulbins, E.; Muehlbacher, M. Functional Inhibitors of Acid Sphingomyelinase (FIASMAs) in E. Gulbins and I. Petrache (eds.), Sphingolipids: Basic Science and Drug Development, Handbook of Experimental Pharmacology 215, DOI 10.1007/978-3-7091-1368-4_9, # Springer-Verlag Wien 2013.

			**median S-ASM activity (pmol/h/µL)**										***p* values (with vs. without drug)**				
**Drugs**	**class**	**FIASMA**	**all**	***n***	**UP**	***n***	**MP**	***n***	**RP**	***n***	**HC**	***n***	**all**	**UP**	**MP**	**RP**	**HC**
all			161.8	229	150.5	63	175.7	66	150.4	39	173.2	61					
no drugs			151.8	101	148.6	41		0	144.8	17	152.4	43					
any drug			175.7	128	150.9	22	175.7	66	154.4	22	202.4	18	**0.018**	0.594		0.380	**0.006**
anti-epileptic drug (AED)	1		156.4	10	216.3	2	156.4	6	144.0	2	.	0	0.281		0.969		0.075
antipsychotic	2		173.7	33	219.7	3	170.0	29	177.2	1	.	0	0.291	0.322	0.713	0.183	
benzodiazepine	3		137.8	14	219.7	3	137.7	11	.	0	.	0	0.909		0.655		
monoamine oxidase inhibitor (MAOI)	4		262.8	3	.	0	262.8	3	.	0	.	0	0.649		0.662		
noradrenergic and specific serotonergic antidepressant	5		185.7	23	91.5	1	190.7	22	.	0	.	0	0.698		0.705	0.183	
norepinephrine and dopamine reuptake inhibitor	6		164.5	7	.	0	160.2	6	177.2	1	.	0	0.137		0.305	0.425	
selective serotonin reuptake inhibitor (SSRI)	7		181.9	36	216.3	2	184.1	28	129.5	6	.	0	0.813		0.574	0.656	
serotonin and norepinephrine reuptake inhibitor	8		141.3	18	.	0	141.3	16	138.3	2	.	0	0.269		0.358		
stimulant	9		276.7	1	.	0	276.7	1	.	0	.	0	0.215		0.520	0.374	
tricyclic antidepressant (TCA)	10		137.9	11	.	0	131.3	10	186.0	1	.	0	0.211		0.463	0.790	
different psychotropic drug	11		205.4	12	91.5	1	206.4	11	.	0	.	0	0.452	0.187	0.348		
Acetylsalicylic acid	-	-	254.2	2	223.3	1		0		0	285.1	1	0.081	0.296		0.173	
Aconit pain relieving plant oil	-		187.1	1		0	187.1	1		0		0	0.535		0.694		
Agomelatine	11		187.2	3		0	187.2	3		0		0	0.362		0.569		
Allopurinol	-		207.3	2		0	101.3	1		0	313.3	1	0.780		0.198		0.125
Alprazolam	3		87.0	1	87.0	1		0		0		0	0.151	0.138			
Amiloride	-		224.4	2	135.5	1		0		0	313.3	1	0.493	0.700			0.125
Amisulpride	2		211.6	1		0	211.6	1		0		0	0.364		0.416		
Amitriptyline	10	+	137.7	1		0	137.7	1		0		0	0.586		0.416		
Amlodipine	-	+	313.3	1		0		0		0	313.3	1	0.123				0.120
Aripirazole	2		114.0	3		0	114.0	3		0		0	0.528		0.380		
Asenapine	2		164.5	1		0	164.5	1		0		0	0.928		0.813		
asthma spray	-		184.8	1		0		0		0	184.8	1	0.576				0.650

Atorvastatin	-		155.8	2		0	101.3	1	210.4	1		0	0.855		0.198	0.248	
Bisoprolol	-		151.3	3	246.2	2		0	103.1	1		0	0.888	0.240		0.424	
Bisphosphonate	-		345.3	1		0		0		0	345.3	1	0.102				0.100
Budesonide	-		151.3	1	151.3	1		0		0		0	0.809	0.956			
Bupoprion	6	-	174.5	7		0	174.1	6	219.2	1		0	0.698		0.705	0.183	
Candesartan	-		118.8	5		0	118.8	3	162.6	2		0	0.785		0.678	0.610	
Carbamazepine	1	-	174.5	3		0	174.5	3		0		0	0.986		0.747		
Carvedilol	-	+	276.7	1		0	276.7	1		0		0	0.155		0.135		
Cetirizine	-		241.6	2	.	0	241.6	2	.	0	.	0	0.108		0.108		
Citalopram	7	-	162.5	12		0	155.8	9	206.1	3		0	0.785		0.336	0.562	
Clomipramine	10	+	118.8	1		0	118.8	1		0		0	0.364		0.331		
Clozapine	2	-	100.0	1		0	100.0	1		0		0	0.232		0.181		
Diazepam	3	-	145.4	1		0	145.4	1		0		0	0.717		0.582		
Duloxetine	8		170.2	8		0	176.9	7	112.8	1		0	0.888		0.763	0.594	
Enalapril	-		187.2	3		0	187.2	3		0		0	0.629		0.914		
Escitalopram	7		211.6	9		0	211.6	9		0		0	0.240		0.317		
Ezetimibe	-		61.3	1	61.3	1		0		0		0	0.096	0.099			
Flurazepam	3		211.6	1		0	211.6	1		0		0	0.364		0.416		
Fluticason/Salmeterol	-	-	101.3	1		0	101.3	1		0		0	0.250		0.198		
Homeopathic drugs	-		205.5	2	205.5	2		0		0		0	0.966	0.969			
Hormonal contraceptive	-		184.5	13	112.5	2	375.9	1	141.0	2	187.7	8	0.626	0.196	0.098	0.799	0.455
Hydrochlorithiazide	-		165.6	6	188.8	2	157.3	2	112.8	1	313.3	1	0.717	0.583	0.736	0.594	0.125
Ibuprofen	-	-	104.5	1		0	104.5	1		0		0	0.263		0.217		
Insulin	-		150.5	3	150.5	1	179.6	2	.	0	.	0	0.191	1.000	1.000		
Iodine	-		155.4	5	162.8	2	14.1	1	163.3	2		0	0.433	0.666	0.088		0.610
Irbesartan	-		313.3	1		0		0		0	313.3	1	0.123				0.125
Lamotrigine	1	-	173.3	4		0	145.4	3	210.4	1		0	0.790		0.633	0.248	
Lercanidipine	-	-	153.0	2		0	153.0	2		0		0	0.847		0.708		
Levodopa/benserazide	11		246.2	1		0	246.2	1		0		0	0.244		0.306		
Lisinopril	-		242.1	1	242.1	1		0		0		0	0.257	0.248			
Lithium	11		170.7	6		0	183.0	5	158.4	1		0	0.774		0.913	0.790	
Loratadine	-	+	187.8	2	187.8	2		0		0		0	0.676	0.530			
Lorazepam	3		177.4	12	230.9	2	144.4	10	.	0	.	0	0.757	0.126	0.128		
Losartan	-		389.2	1	389.2	1		0		0		0	0.090	0.088			
L-Thyroxin	-		180.4	34	147.0	6	183.0	13	194.0	10	223.8	5	0.195	0.623	0.968	**0.029**	0.153
Macrogol	-		100.0	1		0	100.0	1		0		0	0.232		0.181		
Magnesium	-		150.4	1		0		0	150.4	1		0	0.785			1.000	
Metformin	-		169.0	2		0	118.8	1	219.2	1		0	0.966		0.331	0.183	
Metoprolol	-		212.0	2		0	211.6	1	212.5	1		0	0.195		0.416	0.214	
Mirtazapine	5	-	176.9	23	.	0	176.9	23	.	0	.	0	0.649		0.662		

Moclobemide	4		128.1	2		0	128.1	2		0		0	0.379		0.246		
Modafinil	9		240.3	1		0	240.3	1		0		0	0.269		0.358		
Nifedipine	-		273.9	1		0	273.9	1		0		0	0.164		0.149		
Nitrendipine	-		183.5	1		0	183.5	1		0		0	0.628		0.854		
Nortriptyline	10	+	165.6	2		0	165.6	2		0		0	0.881		0.881		
Olanzapine	2		164.5	5		0	164.5	5		0		0	0.865		0.490		
Opipramol	10	-	229.8	4		0	273.6	3	186.0	1		0	0.103		0.144	0.374	
Oxcarbazepine	1		196.3	1		0	196.3	1		0		0	0.468		0.582		
Pancreatin	-		225.6	1		0		0	225.6	1		0	0.304			0.155	
Pantoprazol	-		118.8	5	135.3	2	100.0	3		0		0	**0.040**	0.505	**0.030**		
Paroxetine	7	+	273.9	1		0	273.9	1		0		0	0.164		0.149		
Pipamperone	2	-	185.4	6	219.7	1	183.5	5	.	0	.	0	0.635	0.322	0.837		
Pregabalin	1		196.9	4		0	174.5	3	219.2	1		0	0.311		0.794	0.183	
Propranolol	-	-	163.6	1		0	163.6	1		0		0	0.988		0.733		
Quetiapine	2	-	164.5	11		0	164.5	11		0		0	0.453		0.763		
Ramipril	-		179.0	12	150.3	3	179.0	6	161.1	2	285.1	1	0.232	0.540	0.698	0.702	0.173
Risperidone	2		177.9	6		0	170.0	5	219.2	1		0	0.382		0.990	0.183	
Salbutamol	-		212.5	3		0	187.6	2	212.5	1		0	0.499		0.911	0.214	
Selen	-		155.3	1		0		0		0	155.3	1	0.892				0.776
Sertraline	7	+	184.7	11		0	184.7	9	165.0	2		0	0.218		0.355	0.610	
Simvastatin	-		220.0	2		0		0	126.7	1	313.3	1	0.555			0.790	0.125
Sitagliptin	-		242.1	1	242.1	1		0		0		0	0.257	0.248			
St John's wort	11		187.1	1		0	187.1	1		0		0	0.535		0.694		
Statins	-		137.7	1		0	137.7	1		0		0	0.586		0.416		
Sumatriptan	-		164.6	2	164.6	2		0		0		0	0.829	0.811			
Tamoxifen	-	+	264.1	1		0	264.1	1		0		0	0.180		0.198		
Tamsulosin	-		389.2	1	389.2	1		0		0		0	0.090	0.088			
Tianeptine	10		225.9	2		0	225.9	2		0		0	0.154		0.217		
Tranylcypromine	4		227.6	1		0	227.6	1		0		0	0.297		0.386		
Trimipramine	10	+	163.6	1		0	163.6	1		0		0	0.988		0.733		
Urapidil	-		118.8	1		0	118.8	1		0		0	0.364		0.331		
Valproic Acid	1		164.5	1		0	164.5	1		0		0	0.940		0.773		
Valsartan	-		135.7	2		0	101.3	1		0	170.2	1	0.486		0.198		0.955
Venlafaxine	8	-	184.1	12		0	183.5	11	210.4	1		0	0.677		0.673	0.248	
Vitamine B4	-		73.5	1		0		0	73.5	1		0	0.166			0.131	
Vitamine B6	-		112.0	2		0		0	112.0	2		0	0.972			0.279	
Vitamine D	-		114.8	6		0	91.8	3	139.8	2	213.8	1	0.162		**0.024**	0.750	0.394
Zinc	-		105.9	2	61.3	1		0	150.4	1		0	0.170	0.099			1.000
Ziprasidone	2		174.5	1		0	174.5	1		0		0	0.809		0.979		

Supplemental table S4. Comparison of activities of secretory acid sphingomyelinase (S-ASM) in the serum of patients and controls depending on comorbidities and smoking status. Comorbidities were assessed according to the structured clinical interview for DSM-IV. Groups: PU unmedicated depressive patients, PM medicated depressive patients, HC healthy controls, PR patients with remitted major depressive disorder; Mann–Whitney U-test and Kruskal-Wallis test with nominal $p < 0.05$ in bold. The following categories were completely absent in our cohort: schizophrenia, schizophreniform disorder, substance abuse (alcohol or other drugs), obsessive-compulsive disorder, posttraumatic stress disorder, somatization disorder.

		**median S-ASM activity (fmol/h/µL)**										***p* value**				
Comorbidities		**all**	***n***	**PU**	***n***	**PM**	***n***	**PR**	***n***	**HC**	***n***	**all**	**PU**	**PM**	**PR**	**HC**
Smoking status	smoker	189.1	56	153.2	16	187.2	23	212.5	7	192.2	10	**0.011**	0.950	0.163	**0.036**	0.224
	ex-smoker	155.3	49	143.4	14	146.0	9	111.8	10	176.4	16					
	nonsmoker	153.7	124	150.5	33	165.4	34	151.8	22	152.4	35					
Bipolar I	yes	164.5	11	157.2	4	164.5	7	.	0	.	0	0.978	0.800	0.860		
	no	161.2	218	150.5	59	180.8	59	150.4	39	173.2	61					
Bipolar II	yes	206.1	4	135.5	3	276.7	1	.	0	.	0	0.433	0.897	0.135		
	no	161.8	225	150.9	60	174.5	65	150.4	39	173.2	61					
Panic disorder	yes	151.3	15	151.3	9	153.8	6	.	0	.	0	0.672	0.844	0.365		
	no	163.6	213	150.4	54	180.8	59	150.4	39	173.2	61					
Agoraphobia. social phobia. specific phobia. generalized anxiety disorder	yes	179.3	33	178.3	14	184.7	15	154.3	3	163.7	1	0.066	0.120	0.134	0.685	0.865
	no	155.5	193	148.6	49	170.0	49	144.8	35	173.6	60					

Supplemental table S5. Correlation between S-ASM activity and change of the 20 sub-item scores of the STAI trait inventory between inclusion and follow-up (approximately three weeks) in medicated female depressed patients ($n = 32$, rho and p values from Spearman correlations, nominal $p < 0.05$ in bold).

**item #**	**1**	**2**	**3**	**4**	**5**	**6**	**7**	**8**	**9**	**10**	**11**	**12**	**13**	**14**	**15**	**16**	**17**	**18**	**19**	**20**
reversed	x					x	x			x			x			x			x	
rho	**0.474**	-0.088	-0.081	-0.073	0.110	**0.434**	**0.575**	0.058	0.119	**0.497**	0.159	0.126	**0.409**	0.208	0.038	**0.540**	0.242	0.100	**0.450**	0.105
p	**0.006**	0.631	0.658	0.693	0.550	**0.013**	**0.001**	0.751	0.515	**0.004**	0.383	0.493	**0.020**	0.254	0.837	**0.001**	0.183	0.587	**0.010**	0.567
